# Supplementary material for: Barriers to timely disclosure of HIV serostatus: A qualitative study at care and treatment centers in Dar es Salaam, Tanzania
Source: PLoS One. 2021 Aug 26;16(8):e0256537. doi: 10.1371/journal.pone.0256537 (PMC8389510; doi:10.1371/journal.pone.0256537)
Supplement: S1 File — (DOCX) [file pone.0256537.s001.docx]

# Appendix 5: Interview guide - English


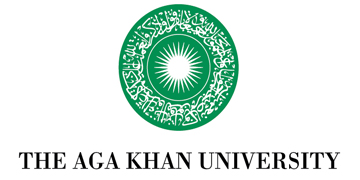


The participant will be in a private room and will be once again ensured of confidentiality and privacy. This is to assure that the participant feels safe, trusts the research team and also feels secure enough to share his/her experience in the process of disclosing of his/her serostatus.

1. Tell me about yourself?
2. When did you find out about your serostatus?
3. Can you describe for me how you felt when you found out about your serostatus?
4. When did you disclose your serostatus
5. Why did you not disclose when you found out? What is it that stopped you? (probe for reasons and barriers)
6. How did you disclose your serostatus? please describe your experience
7. Can you explain what made you decide to disclose to ____ ?(probe for reasons for disclosure)
8. What was the reaction of _____ after disclosure (probe for answers – stigma, isolation, acceptance, no- acceptance, violence, partner testing, breach of confidentiality, impact on relationship)?
9. How are you coping after disclosure? (probe for support (financial, emotional, ART support - ease to access HIV-Related services, ART adherence and retention, use of ART freely), stigma experienced, loneliness, depression, impact on relationship)
10. In your opinion, what makes it difficult for people to disclose?
11. What would help/would facilitate people to disclose their HIV status?
12. I do not have any other questions; do you have anything else that you would like to share with me that I did not ask in relation to the topic?
