# Supplementary material for: Barriers to timely disclosure of HIV serostatus: A qualitative study at care and treatment centers in Dar es Salaam, Tanzania
Source: PLoS One. 2021 Aug 26;16(8):e0256537. doi: 10.1371/journal.pone.0256537 (PMC8389510; doi:10.1371/journal.pone.0256537)
Supplement: S2 File — (DOCX) [file pone.0256537.s002.docx]

# Appendix 6: Interview guide – Kiswahili


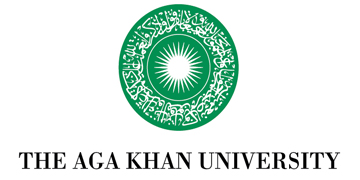


**Mwongozo wamahojiano**

Mshiriki atakua kwenye chumba pekee. Mshiriki atahakikishwa usiri tena.

1. Niambie kuhusu wewe, jitambulishe
2. Je, nilini ulijua hali yako yaambukiziya UKIMWI
3. Je, ulijisikiaje baada yakuweka wazi hali yako yamaambukizi
4. Je, nilini uliweka wazi hali yakoyaambukiziya UKIMWI
5. Je, Kwanini haujaweka wazi mapema hali yako yamaambukizi? Nini ilikusababisha usiwekewazi? (uliza kwa ndani sababu za Vizuizi katika kutoa taarifa zawakati vya yaamubukizi)
6. Je, ulitumia njia gani kuweka wazi hali yako yaambukiziya UKIMWI
7. Je unaweza kunieleza kilichokufanya ukaweka wazi hali yako yaambukizi ya UKIMWI (uliza kwa undani sababu yakuweka wazi hali yaambukiziya UKIMWI)
8. Je, huyo mtua lijisikiaje baada ya kuweka wazi hali yako ya maambukizi? (uliza kwaundani kuhusumajibu –unyanyapaa, kutengwa, kukubalika, kutokubalika, vurugu, mwenzakupimamaambukizi, kuwekawazi, athari)
9. Je, unakukabilianaje na hali yako baada yakuwekawazi hali yako yaambukiziya UKIMWI (uliza kuhusu msaada kifedha, kifikra, dawa za kufumbaza, unynyapaa, upweke, msongo mawazo na athari yakimahusiano)
10. Kwa mawazo wako nivitu gani vinaweka ugumu kuwekawazi hali yamaambukuzi ya UKIMWI
11. Je, ni vitu gani vita wasaidia watu kuweka wazi halizaozamaambukiziya UKIMWI
12. Sina swali lolote, je kuna kitu kingine unataka kuniambia kuhusimadahii
