# Supplementary material for: Barriers to timely disclosure of HIV serostatus: A qualitative study at care and treatment centers in Dar es Salaam, Tanzania
Source: PLoS One. 2021 Aug 26;16(8):e0256537. doi: 10.1371/journal.pone.0256537 (PMC8389510; doi:10.1371/journal.pone.0256537)
Supplement: Attachment — Submitted filename: Response to Reviewers.docx [file pone.0256537.s003.docx]

Barriers to timely disclosure of HIV serostatus: A qualitative study at care and treatment centers in Dar es Salaam, Tanzania.

| Reviewers comment | Response | Page/line number |
| --- | --- | --- |
| Introduction 1. Line 59-61: Authors need to make it clear that disclosure of HIV status is one of the strategies that can help to prevent the transmission of HIV. The way it has been written, it is like disclosure of HIV status is the only way that can help to prevent the transmission of HIV | Agreed and changed | Line 57-59 |
| 2. The sentence covered by lines 65-67 needs citation. | Agreed and added reference | Line 67 |
| 3. It is important to describe the operational definition of HIV disclosure in this study as this can help to guide the interpretation of the findings | Agreed and added in introduction | Line 60-61 |

| 4. The justification for conducting this study is not convincing or correct. Authors identify lack of studies that have assessed barriers to timely disclosure. However, contrary to the authors’ claim there are several studies in Tanzania that have been conducted related to barriers to disclosure of HIV status to others or sexual partners some of them include the following (Damian et al., 2019; Hallberg et al., 2019; Maluka, 2014). Thus, authors need to be clear with the reasons for conducting this study. Why is it important to conduct this study? What will it add to the existing literature regarding disclosure of HIV status to others/sexual partners in Tanzania? | Modified the wording on justification.  There are studies that have shown the prevalence in the delay in disclosure, and some managed to get reasons but they were not explored in depth to get a deeper meaning qualitatively especially in Tanzania.  This study deals with the barriers to and facilitators of motivation for the timely disclosure. It explores the reasons why PLWHA have a delay in disclosing and what helped them eventually overcome it.  Knowing the barriers that prevent timely disclosure will help counsellors address them to promote early disclosure to reap the benefits of timely disclosure such as ARV adherence, viral suppression and prevention of the spread of HIV.  The study by Hallberg et al and Damian et al were quantitative studies that focused on factors that affected disclosure, they did not study reason/barriers for timely disclosure. Study by Maluka too did not study timely disclosure | Line 98-117 |
| --- | --- | --- |

| Materials and methods  5. Where did data collection take place in the research site?  How were participants approached to take part in the interview?  How long did each interview take to be completed?  How many people were involved in the data analysis process? | Modified and added the information | Line 155-157  Line 152-153  Line 159-161  Line 190-192 |
| --- | --- | --- |
| 6. Given that no interview took place at Aga Khan Hospital, it is not necessary for the authors to include information about this facility in this article. | I think it is important to include Aga Khan hospital. Lack of participants explains the social class theory that we mentioned in the discussion section. | Line 459 -473 |
| 7. What type of questions were included in the interview guide. It would be important if authors can include the interview guide as supplementary material to their submission | Uploading the interview guide as supporting information – S1 and S2 | Line 159 |
| Results 8. The findings in the three categories seem to be limited. For example, the last category ‘Consequences of delayed disclosure’ is too brief and lacks sufficient detail to be a standalone category. | Added details in the third category | Line 348 -364 |
| As a qualitative study, the findings of the study should have some depth to enable the readers to understand participants’ perspectives. The findings are also missing some important information that might be relevant to the study aim for example, what prompted participants to disclose their HIV status to the people they disclosed to? | This was addressed in the sub-category motivation for disclose of serostatus where we discussed that participants would disclose to those they were close to and had established trust with them | Line 323 -330 |
| How did the significant others/family members reacted to the disclosure | Included as negative and positive reactions/outcomes in the results section.  Positive reactions were added under the subcategory motivations to disclose. This is because positive reactions encouraged further disclosure.  Negative reactions were placed under the subcategory Barriers hindering timely disclosure as these reactions prevented further disclosure | Line 331 -346  Line 256 - 288 |
| How was the disclosure done? | Not part of my objectives |  |
| Discussion 9. Authors have discussed some findings which are not presented in the findings section. For example, line 316-318, the authors have indicated that a majority of the participants experienced some sort of stigmatization (getting laughed at, discrimination); infidelity and one participant attempted suicide. This information is not described in the findings section. | This is included in the findings under the subcategory barriers to disclose. Experiencing a negative outcome hindered and prevented further disclosure | Line 256-288 |
| The limitations of the study need more work. Aga Khan Hospital is not part of the research site in this study as none of the participants was recruited from this research site. Researchers should highlight the limitations of the research methodology used in this study. | Our limitation was based on the fact that we got no participants from Aga khan. Which was why we discussed social class and perceived stigma – see comment 12 below.  However I have added other limitations after discussion section | Line 459-475 |
| 11. The implications of findings for practice, research and policy are also not clearly explained in this section. | Discussed in the conclusion  Implication of finding: strengthen the counselling services of HCW by training to promote timely disclosure.  Areas of research: stigma surrounding HIV, relationship of social class and HIV | Line 493-497  Line 485-491 |
| 12. Lines 352 to 256, authors have discussed social class in relation to stigma. However, social class has not been presented in the findings of the study. Authors need to discuss or make research recommendations based on the findings of the study. | Social class was brought up when we tried to understand why we were not able to recruit any participant from the Aga Khan hospital which caters for relatively higher class of patients.  Our recommendation was to have other studies that see if there is a relation with HIV stigma and social class. Evidence show that social class in HIV does not receive much notice in literature and is a neglected area in research (see discussion) | Line 464-473 |
| General comment 13. All sections of the paper need editing. | Thank you for your comments and review |  |
| Reviewer #2: Congratulations on an interesting study, the findings of which should be of interest to others working in Africa.  The methods you have used and have described in your paper are sound and appropriate. The results, whilst not new, are important as they highlight the need for consistent high quality counselling of PLWHA in order to disclose early and interrupt onward transmission to sexual partners. The points you raise in your discussion are clear and reference other appropriate and relevant research. I suggest you consider including in the discussion some thoughts about the importance of adherence to ARVs and attaining a suppressed viral load in interrupting onward HIV transmission to susceptible partners - i.e. undetectable viral load = unable to transmit.  From a population perspective, I think this is a vital point that is a consequence of the point you raise about people who are provided appropriate counselling and support,that they are more likely to disclose earlier and also more likely to adhere to treatment.  My main concern with this paper is the style in which it is written. The writing style, in my opinion, is not of an academic standard that would be acceptable to peer reviewed journals and needs to be tightened up considerably. There is a consistent use of 'non academic language' throughout the paper and whilst the content is sound, the presentation is not. e.g. 'What stood out for the most part' - this phrase is fine when speaking but does not read well in an academic paper. Another example, using words such a 'spread' in reference to HIV is not ideal, it would be preferable to use the word 'transmission'. I would encourage you to look closely at the expressions you have used and re-write the paper using acceptable academic phrasing (e.g. analysis was done - would read better as -analysis was undertaken). The grammar also needs some significant work.  There are some font issues - e.g. see lines 99 & 100 Also, line 297 - write vs as a word, i.e. versus | Thank you for your comments  We have included the importance of ARV in the discussion  We also have edited the grammatical and vocabulary errors throughout the paper using academic language and tracked the changes  Corrected the font | Line 381 -385 |
