# Supplementary material for: Barriers to timely disclosure of HIV serostatus: A qualitative study at care and treatment centers in Dar es Salaam, Tanzania
Source: PLoS One. 2021 Aug 26;16(8):e0256537. doi: 10.1371/journal.pone.0256537 (PMC8389510; doi:10.1371/journal.pone.0256537)
Supplement: Attachment — Submitted filename: Response to reviwers.docx [file pone.0256537.s004.docx]

**Barriers to timely disclosure of HIV serostatus: A qualitative study at care and treatment centers in Dar es Salaam, Tanzania.**

| Reviewers comment | Response | Page/line number |
| --- | --- | --- |
| Reviewer #1: The authors have addressed issues identified in the previous review. However, The paper needs thorough editing to make it suitable for publication as there are many typos and grammar errors that are obscuring the clarity of the paper. | Have resubmitted it to English editors to make it ready for publication |  |
| In the conclusion section, page 34 line 489-490 reads, “This paves the way to achieve two parameters of the 90-90-90 target set by UNAIDS; namely, easier access and linkage to medications and subsequent viral suppression.” Authors need to be aware that the 90-90-90 target set by UNAIDS finished in 2020. UNAIDS set another target, the 95:95:95 target to end the AIDS epidemic by 2030 | Thank you for pointing that out. We have rectified it both in the introduction and conclusion. | Line 69-72 and  Line 496-498 |
| Reviewer #2: Thank you for addressing my previous comments.  One thing that surprises me is that none of the participants in your study questioned where they had acquired the HIV infection from - for example - none suspected they had acquired it from their sexual partner and therefore questioned that partner's fidelity. This is a common occurrence in other countries, especially from women who suspect their husband/partner has had sex with another (infected) person. Perhaps you could add a sentence or two about why the participants in your study didn't suspect their partner's of infecting them –  did all participants have more than one sexual partner?  You would also need to comment about whether or not your participants had had a previous negative HIV test and could therefore estimate the period of time in which they became infected. e.g addressing whether Tanzania routinely test antenatal women for HIV?  Otherwise, Congratulations on an interesting paper. | Thank you so much for insightful feedback and comments about our manuscript. This study main aim was to explore the barriers contributing to delayed disclosure and the reasons that made participants overcome it and finally disclose.Since it was not the focus of our study, we did not ask if they questioned where they had acquired the infection from and we do not have data for such.  However, the data shows that, after disclosing to partners they were blamed to have been the ones who brought the disease into the relationship. This led to a strain and in some cases a break in the relationship.  This is mentioned in the barriers of disclosure under the negative outcomes experienced.  From the interviews we did, the participants all had one partner at time of disclosure.  Mentioned in the results section  All participants that were interviewed had not tested prior and so did not have a negative test before that.  Added a couple of lines in the discussion section including the introduction of HIV testing in routine antenatal and RCH services  We appreciate the feedback that has helped improve the quality of our manuscript. | Line 265-281  Line 225-226  Line 225 -226 and 436-438  Line 433 -439 |
