# Supplementary material for: Barriers to timely disclosure of HIV serostatus: A qualitative study at care and treatment centers in Dar es Salaam, Tanzania
Source: PLoS One. 2021 Aug 26;16(8):e0256537. doi: 10.1371/journal.pone.0256537 (PMC8389510; doi:10.1371/journal.pone.0256537)
Supplement: Attachment — Submitted filename: Response to reviwers.docx [file pone.0256537.s005.docx]

**Barriers to timely disclosure of HIV serostatus: A qualitative study at care and treatment centers in Dar es Salaam, Tanzania.**

| Reviewers comment | Response | Page/line number |
| --- | --- | --- |
| Reviewer #1: The authors have addressed the comments I made in my previous review. Nonetheless, there are still several areas in the manuscript where there is no spacing between words or between words and references e.g. lines 52, 57, 51, 89, 172. Line 85 has a different font size from the rest of the manuscript. | Thank you for your observation.  We have rectified all the spacing issues and fonts. | Multiple places all marked on the “manuscript with tracked changes” document. |
